# Glycosyltransferases Expression Changes in *Leuconostoc mesenteroides* subsp. *mesenteroides* ATCC 8293 Grown on Different Carbon Sources

**DOI:** 10.3390/foods12091893

**Published:** 2023-05-04

**Authors:** Luz Cristina Vallejo-García, María del Carmen Sánchez-Olmos, Rosa María Gutiérrez-Ríos, Agustín López Munguía

**Affiliations:** 1 Departamento de Ingeniería Celular y Biocatálisis, Instituto de Biotecnología, UNAM, Av. Universidad 2001, Col. Chamilpa, Cuernavaca 62210, Morelos, Mexico; cristina.vallejo@ibt.unam.mx; 2 Departamento de Microbiología Molecular, Instituto de Biotecnología, UNAM, Av. Universidad 2001, Col. Chamilpa, Cuernavaca 62210, Morelos, Mexicorosa.gutierrez@ibt.unam.mx (R.M.G.-R.)

**Keywords:** *Leuconostoc mesenteroides*, glycosyltransferases, regulation, sucrose, fructose, xylose

## Abstract

*Leuconostoc mesenteroides* strains are common contributors in fermented foods producing a wide variety of polysaccharides from sucrose through glycosyltransferases (GTFs). These polymers have been proposed as protective barriers against acidity, dehydration, heat, and oxidative stress. Despite its presence in many traditional fermented products and their association with food functional properties, regulation of GTFs expression in *Ln. mesenteroides* is still poorly understood. The strain *Ln. mesenteroides* ATCC 8293 contains three glucansucrases genes not found in operons, and three fructansucrases genes arranged in two operons, *levLX* and *levC*-*scrB*, a Glycoside-hydrolase. We described the first differential gene expression analysis of this strain when cultivated in different carbon sources. We observed that while GTFs are expressed in the presence of most sugars, they are down-regulated in xylose. We ruled out the regulatory effect of CcpA over GTFs and did not find regulatory elements with a direct effect on glucansucrases in the condition assayed. Our findings suggest that only operon *levLX* is repressed in xylose by LexA and that both fructansucrases operons can be regulated by the VicK/VicR system and PerR. It is essential to further explore the effect of environmental conditions in *Ln. mesenteroides* bacteria to better understand GTFs regulation and polymer function.

## 1. Introduction

*Leuconostoc mesenteroides* bacteria are Gram-positive, catalase-negative, non-motile, non-sporogenous, and facultative anaerobic often found as coccoid shape groups arranged in pairs or chains [1]. They may be found in plant roots, vines, and leaves, from where they are transferred to utensils, animal milk, fruits, and vegetables [2]. *Ln. mesenteroides* has a heterofermentative metabolism that leads mainly to lactic acid, carbon dioxide, and ethanol, but also to products such as acetate, acetoin, diacetyl, or 2,3-butylene glycol, important components of flavor in fermented products [3].

One of the most interesting properties of *Ln. mesenteroides* strains is their ability to produce polysaccharides when growing in sucrose-rich environments such as sugar mills. The first *Ln. mesenteroides* strain was isolated from a gelatinous substance commonly observed over machinery surfaces in sugar mills [4]. In the early 20th century, this substance was identified as dextran [5], a glucose homopolysaccharide (HoP). The discovery of the enzymatic nature of dextran synthesis [6] and the application of dextran as a plasma substitute [7] increased the interest in the search for adequate strain/enzyme sources and the optimization of reaction conditions for dextran synthesis. As a consequence, *Ln. mesenteroides* NRRL B512F was isolated, selected and classified as “the industrial strain”; both the strain and the responsible enzyme have since then extensively studied [8,9,10,11].

Besides dextran, various *Ln. mesenteroides* strains are potential producers of fructose HoPs (levan or inulin) as in the case of the industrial strain B512F that contains an active fructansucrase (producing levan) in its genome [12]. After a search in the GenBank NCBI database [13], we found around 150 sequenced genomes of *Ln. mesenteroides* strains. Within this group, it was possible to discover strains bearing several genes coding for GTFs: two, for instance, in strains B512F and Lm17; three in the strain URE13; four in the strains P45 and SRCM103460; and five in strains such as KFRI-MG, WiKim32, and CBA7131 among many others.

The biological role of HoPs in *Ln.* strains is not sufficiently clear, though the existing hypothesis points out functions such as energy storage, environmental protection (adverse pH, desiccation, depredation), cell adhesion (oral cavity or intestinal tract), and cell communication in biofilms [14,15,16,17,18]. Moreover, despite the increasing evidence of the role of *Ln. mesenteroides* HoPs as food nutraceuticals and their wide structural diversity, the interest in regulation is scarce and mainly focused on *Streptococcus mutans* GTFs, where HoPs are considered a pathogenic trait given their role in bacteria colonization on teeth [19,20]. However, regulation studies regarding the expression of GTFs in Lactic Acid Bacteria (LAB) could shed light on the environmental and biological role of HoPs, and their functional role in fermented foods in particular. Early research on regulation in *Ln. mesenteroides* strains demonstrated a decrease in GTFs activity when glucose or fructose are used instead of sucrose as a carbon source in aerobic cultures [21,22,23]. The presence of diverse mechanisms for the uptake of carbon sources, such as permeases and the involvement of the Phosphotransferase System (PTS) was made evident [18,23,24], and most conclusions indicated that sucrose is an atypical inducer for dextransucrase, at least in the case of *Ln. mesenteroides* NRRL B512F where the existence of post-transcriptional regulation was suggested [22].

*Ln. mesenteroides* subsp. *mesenteroides* ATCC 8293 (equivalent to NRRL B-1118, DSM 20343, NCTC 12954, NCDO 523) is a GRAS (Generally Recognized As Safe) containing six identified GTFs genes corresponding to a 308 kDa glucansucrase (DsxD); a 166 kDa glucansucrase (DsrD) identical to *Ln. mesenteroides* NRRL B512F dextransucrase; a 162 kDa glucansucrase (DsrI) producing insoluble dextran [25]; two fructansucrases characterized after heterologous expression (109 kDa LevC and 108 kDa LevL, both producing levans), [26]; and finally, a non-characterized 109 kDa fructansucrase (LevX). The strain was isolated from fermented olives and their annotated genome was the first one available [27]. Therefore, this strain has served to identify the presence of *Ln. mesenteroides* strains in fermented products such as kimchi, sauerkraut, pulque, kefir, pozol, and several fermented fruit juices, and have been the reference for metagenomic analysis of vegetable fermented foods [28,29,30,31].

Depending on their carbohydrate composition, type of linkage, and branching, as well as molecular mass, HoPs have physicochemical properties adequate for industrial application as emulsifiers, thickeners, stabilizers, sweeteners, and/or excipients. In particular, levans and inulins also behave as soluble fiber and prebiotics, a function strongly dependent on polysaccharide structure and molecular weight [32,33,34,35]. HoPs have antioxidant and antitumoral properties and can stimulate the immunological system of mammals, also constituting an aid to decrease cholesterol levels [14,15,17,34,36,37].

Traditional fermented foods and beverages have gained increasing importance due to the recognized health benefits delivered by their nutrients and bioactive compounds, including their pre- and pro-biotic content [38]. HoPs characteristics and properties can contribute to these benefits [39], but their synthesis in fermented products is not always assured. The potential of *Ln. mesenteroides* strains to produce a broad spectrum of HoPs structures derived from their GTFs diversity has not received sufficient attention, especially in traditional fermented foods and beverages or in the design of modern fermentation processes. The modulation of GTF expression in the traditional process or a strategical manipulation of environmental factors, particularly the carbon source, may lead to fermented products with a specific or enriched HoP profile. In this paper, we first analyze the effect of different carbon sources in GTFs expression from *Ln. mesenteroides* subsp. *mesenteroides* ATCC 8293 to later explore possible regulation pathways when using fructose or xylose compared to sucrose as carbon source using transcriptomics.

## 2. Materials and Methods

### 2.1. Reagents

Sucrose, D-(−)-fructose, D-(+)-glucose, D-(+)-xylose, D-(+)-galactose, D-(+)-mannose, D-(+)-cellobiose, Folin–Ciocalteu′s phenol reagent, albumin from bovine serum (BSA), KNaC_4_H_4_·4H_2_O, NaOH, Na_2_CO_3_, CuSO_4_, and 3,5-Dinitrosalicylic acid were purchased from Sigma-Aldrich (St. Louis, MO, USA). The electrophoresis reagents were from Bio-Rad (Hercules, CA, USA). K_2_HPO_4_, MgSO_4_, HCl, CaCl_2_, NaCl, MnSO_4_, and FeSO_4_ were from J.T. Baker (S.A. de C.V., Toluca, Estado de México, México) Bacto Yeast Extract from BD Biosciences (San Jose, CA, USA).

### 2.2. Aerobic Culture Conditions

Leuconostoc medium was composed of: yeast extract 20 g/L, K_2_HPO_4_ 20 g/L, MgSO_4_ 0.2 g/L, CaCl_2_ 0.05 g/L, NaCl 0.01 g/L, MnSO_4_ 0.01 g/L, and FeSO_4_ 0.01 g/L. The pH of the culture medium was adjusted to 6.9 by adding HCl. Carbon sources were supplemented from filter-sterilized solutions at 59 mM final concentration in the culture medium. Freeze-dried *Ln. mesenteroides* ATCC 8293 cells were recovered in LM medium with 59 mM of sucrose at 28 °C and 200 rpm in a Jeio-Tech IST-3075 shaker incubator (Lab Companion, Daejeon, Republic of Korea) overnight and then diluted to 0.3 OD in fresh medium with sucrose until mid-exponential phase to prepare glycerol (40% *v*/*v* final) stocks that were stored at −80 °C until usage. To modify the carbon source (CS), 200 µL of glycerol stock was plated in Petri dishes with 59 mM of the new CS and incubated at 28 °C in a 10–140 Incubator (Quincy Lab Inc., Burr Ridge, IL, USA). The resulting colonies were repeatedly streaked in fresh Petri dishes with the new CS. Once the strain was adapted to the new CS, 50 mL of culture medium in 250 mL Erlenmeyer flasks were inoculated with the colonies and incubated overnight at 28 °C and 200 rpm in the shaker incubator with 59 mM of the new CS. Next, the culture was diluted to 0.3 OD in 50 mL of fresh medium and cultured again until the start of the stationary phase. Cells were then centrifuged in Eppendorf tubes for 5 min at 13,000× *g* and 4 °C (FC5515R Ohaus centrifuge, Gießen, Germany) and washed three times with 50 mM pH 6.0 acetate buffer. Fresh cells were used for RNA extraction. Cells were frozen and stored at −20 °C for further analysis. Stored cells were thawed in ice and homogenized in 50 mM pH 6.0 acetate buffer for protein and activity determination. When sucrose was used as CS, dextranase (ENZIQUIM, Mexico City, Mexico) was added to the culture in the last step to facilitate sample processing. The cultures were carried out in triplicate.

### 2.3. Anaerobic Culture Conditions

The cultures in anaerobic conditions were carried out in serum flasks with LM medium. The oxygen in the headspace and the medium was replaced by bubbling each phase with a gas mixture (80% N_2_, 20% CO_2_) for at least 30 min and immediately sealed with rubber stoppers and aluminum seals. The closed flasks were sterilized by autoclaving at 116 °C for 26 min. A sucrose solution of 1.75 M was prepared and micro-filtered with a 0.22 µm syringe filter (Millex, Bad Langensalza, Germany) under aseptic conditions; then, CO_2_ was bubbled to displace oxygen (1 min/mL in the culture medium and 1 min/mL headspace) and immediately sealed with rubber septa and aluminum cap. The sucrose solution was supplemented to the flasks using a sterile syringe to reach 59 mM concentration in the culture medium. The first sealed flask was inoculated from an aerobic culture using sterile syringes, subsequent cultures were inoculated from the immediate last sealed flask. Four serial cultures in sealed flasks were carried out to minimize O_2_ interference. When the culture reached the stationary phase, a fresh anaerobic flask was inoculated and the operation was repeated once a 0.3 OD was reached. After the fourth culture, cells were centrifuged as previously described and frozen at −20 °C for further analysis. All flasks were incubated at 28 °C and 200 rpm. The cultures were carried-out in triplicate.

### 2.4. Biomass and pH Determination

Cell growth was followed by measuring optical density at 600 nm (OD600) using a Beckman DU 640 spectrophotometer (Beckman, Brea, CA, USA), a change of 1 unit of optical density at 600 nm was equivalent to 0.44 g of dry matter. The pH changes were followed by measuring 1 mL of culture with pH Lab Meter Model 827 (MetrOhm, Riverview, FL, USA).

### 2.5. Protein Determination

Protein in cell samples was quantified using the Lowry method [40] with modifications. Initially, the Biuret reaction involves the reduction of copper (Cu^2+^ to Cu^+^) by proteins in alkaline solutions, followed by the enhancement stage, the reduction of the Folin–Ciocalteu reagent (phosphomolybdate and phosphotungstate). After incubation with Folin–Ciocalteu′s phenol reagent, samples were centrifuged for 5–10 s and then absorbance was read at 595 nm using a Beckman DU 640 spectrophotometer (Beckman, Brea, CA, USA). The assay was performed in duplicate for each sample.

### 2.6. GTFs Activity Determination

The total transglycosidase activity was quantified following the initial rate of reducing power release with the 3,5-dinitrosalysilic acid method (DNS) [41]. Total activity refers to enzyme activity when using whole harvested cells, including fructansucrase and glucansucrase activity. Determinations were performed by triplicates for each of the three different flask cultures. One unit of GTFs activity was defined as the amount of enzyme that released 1 µmol of reducing sugars (fructose or glucose) per minute. The specific activity of each sample was determined by calculating the rate of total activity per mg of total protein (U/mg total protein). One-way ANOVA with Tukey’s test with α < 0.05 was used to determine the significative difference between specific activities using Microsoft Excel 2019 Software.

### 2.7. Zymograms

GTFs activity were detected by in situ production of polymers on gel after SDS-PAGE as previously described [42]. Briefly, SDS-PAGE gels were prepared at 10% as described by Laemmli [43] and completed in a Mighty Small electrophoresis chamber (Amersham Biosciences, Amersham, UK). Samples were mixed with SDS sample buffer minus β-mercaptoethanol and applied to gel wells. Gels were charged with 200 µg of protein. After electrophoretic separation of the proteins, the lanes containing the molecular mass reference were cut and stained with GelCode (Thermofisher, Hanover Park, IL, USA) following the manufacturer’s instructions. For in situ detection of GTFs activity, the SDS the lanes containing the sample protein fractions were washed three times with 50 mM pH 6.5, phosphate buffer containing 1% Tween 80 (Merck, Darmstadt, Germany), for protein renaturation, and once with the Tween-free buffer. The renatured gel was incubated in a buffer solution with 100 g/L of sucrose overnight at 28 °C. Then, the gels were incubated for 30 min in a 75% ethanol solution, followed by incubation for 1 h in 0.7% periodic acid (Sigma-Aldrich, St Louis, MO, USA) and 5% acetic acid (Mallinckrodt, Staines-Upon-Thames, Surrey, UK) water solution. Next, the gels were washed three times with 0.2% sodium metabisulfite (J.T. Baker S.A. de C.V., Estado de México, Mexico) and 5% acetic acid solutions for 20 min and finally exposed to the Schiff’s reagent (Sigma-Aldrich, St. Louis, MO, USA) to stain the polymer produced.

### 2.8. RNA-seq Analysis

RNA-seq analysis was performed with three biological replicates for each carbon source. RNA extraction, quality analysis, and sequencing were performed at UUSMB (Unidad Universitaria de Secuenciación Masiva y Bioinformática de la UNAM) facility. The RNA extraction was performed from three biological replicates with the Quick-RNA miniprep kit (Zymo Research, Irvine, CA, USA) following the manufacturer’s protocol from 10 mL of freshly harvested cells. After the integrity and quality analysis were performed (Bioanalyzer 2100, Agilent, Santa Clara, CA, USA) the RNA library was prepared with a Zymo-Seq Ribo Free Total RNA Library kit (Zymo Research) following the manufacturer’s protocol. Sequencing was performed in an Illumina Genome Analyzer II (Illumina, San Diego, CA, USA). We obtained two files from each sample.

### 2.9. Differential Gene Expression Analysis

The RNA libraries were analyzed and cleaned using the fastp software (v.0.21.0) [44]. The Salmon software (v.1.5.1) [45] was employed to map the data against the *Ln. mesenteroides* ATCC 8293 complete genome (GenBank assembly accession: GCA_000014445.1). Differential Gene Expression was determined with the DESeq2 library from R software (v.4.0.0) [46], with sucrose as the control condition. Abundances available in Supplementary Material File S1 and File S2.

### 2.10. Operon Search

The complete genome of *Ln. mesenteroides* ATCC 8293 (GCA_000014445.1) was analyzed with Operon mapper [47] web server (https://biocomputo.ibt.unam.mx/operon_mapper, accessed on 6 June 2022) to obtain a list of the operons in the genome.

### 2.11. Motif Search

From the *Ln. mesenteroides* ATCC 8293 complete genome (GenBank assembly accession: GCA_000014445.1), we extracted the intergenic upstream region of each GTF gene from the start site using the Artemis software (v.18.2.0) [48]. From the extracted upstream regions, we searched the binding site motifs of the regulatory elements CcpA, LexA, and VicR using the FIMO [49] tool from MEME-suite (v.5.4.1) [50], with the default parameters. The CcpA binding site sequence of *Lactobacillaceae* WTGWAARCGYTTWCAW (from the regulon of CcpA in https://regprecise.lbl.gov/regulon.jsp?regulon_id=34354, consulted on 18 September 2022, where W is A or T and H is A, T, or C) was used as input motif. In the case of the LexA binding site, the regulatory sequences from the regulon of LexA for *Ln. mesenteroides* subsp. *mesenteroides* ATCC 8293 (https://regprecise.lbl.gov/regulon.jsp?regulon_id=46374, consulted on 18 September 2022) were used as input motifs. Finally, for VicR, the conserved binding site TGTWAHNNNNNTGTWAH [51], where W is A or T and H is A, T, or C as in *gtfC* promoter in *Strep. mutans* U159 was used as the input motif.

### 2.12. RT-qPCR

RNA extraction, cDNA synthesis, and RT-qPCR assays were performed as previously reported [52] with some variations. Five milliliters of cultures in the early stationary phase in each carbon source were centrifuged to harvest fresh cells, and RNA was isolated from the fresh cells using Trizol reagent (Sigma-Aldrich, St. Louis, MO, USA), following the manufacturer’s instructions. RNA integrity was verified by electrophoresis and the concentration was assessed using a NanoDrop2000 spectrophotometer (Thermo Fisher Scientific, Waltham, MA, USA). To remove DNA contamination, the RNA samples were incubated with RNase-free DNase (1 U/µL; Roche, Basel, Switzerland) for 1 h at 37 °C as indicated by the manufacturer. Complementary DNA (cDNA) was synthesized using Thermo Scientific RevertAid Reverse Transcriptase (200 U/µL, Thermo Scientific, Waltham, MA, USA), with 200 ng of DNA-free RNA as a template and following the manufacturer’s instructions. RT-qPCR assays were performed using Maxima SYBR Green/ROX qPCR Master Mix (2X) (Thermo Scientific, Waltham, MA, USA), in a real-time PCR system (QuantStudio 5; Applied Biosystems, Waltham, MA, USA) following the thermal program: an initial 95 °C for 10 min cycle, 40 cycles of 95 °C for 15 s, and a final 60 °C for 60 s cycle. The *dnaX* gene was used for normalization. Primers sequences for GTFs and *dnaX* are summarized in Supplementary Materials Table S1. The relative expression values were calculated using the geometric mean of Ct in the ∆∆Ct method [53] using Microsoft Excel 2019 Software. This analysis was performed with three biological replicates for each carbon source and by triplicate. Data available in the Supplementary Materials File S3 and File S4.

## 3. Results and Discussion

### 3.1. Specific Total Activity Decreases in CS Different from Sucrose

**Figure 1:**
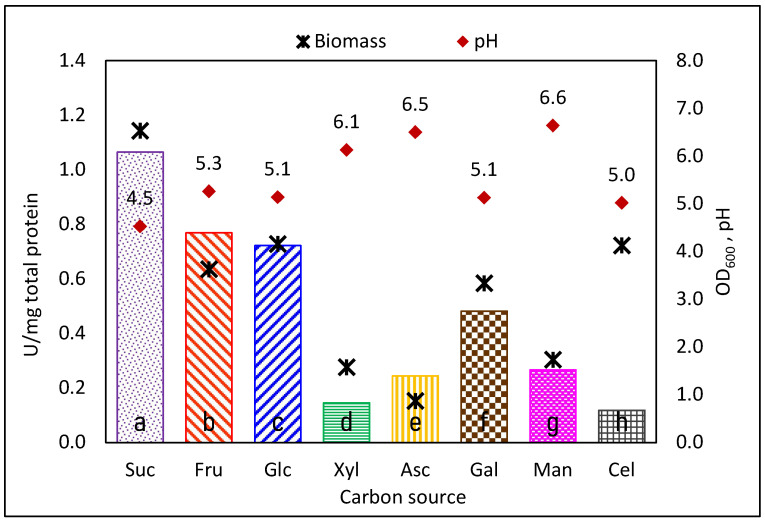
Total-specific GTF activity (bars) found in *Ln. mesenteroides* ATCC 8293 cultures in stationary phase after growth in different carbon sources. The biomass (OD_600_) reached is also reported (✴). Suc = sucrose; Fru = fructose; Glc = glucose; Xyl = xylose; Asc = ascorbic acid; Gal = galactose; Man = mannose; Cel = cellobiose. Different letters in the base of the bars indicate Significant Differences in total activity (ANOVA test with α = 0.05).

We first analyzed the production of GTFs when *Ln. mesenteroides* ATCC 8293 was cultured in several carbon sources: sucrose, commonly accepted as the inductor [22,34], glucose, and fructose. We expanded the study to alternative carbon sources that the strain can metabolize such as xylose, galactose, mannose, cellobiose, and ascorbic acid, since these compounds are common in plants and therefore, eventual CS in natural raw materials fermentation. As expected, the CSs were consumed with different specificities, as concluded from the different biomass yields (OD_600_) reached in the stationary phase (Figure 1). We compared the level of total specific GTF activity produced in each CS, observing a significative decrease when carbon sources other than sucrose were used in the culture medium (Tukey’s test with α = 0.05). As already reported for *Ln. mesenteroides* [21,22,26], sucrose is not the exclusive inductor for GTFs expression, and considerable enzyme activity is detected when the strain is cultured in alternative carbon sources.

**Figure 2:**
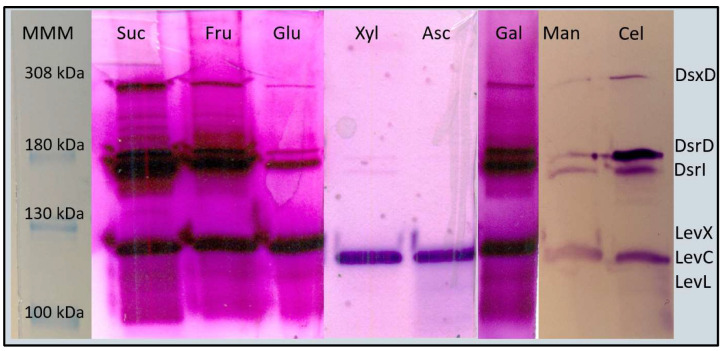
GTF induction as observed by activity zymograms of *Ln. mesenteroides* ATCC 8293 cell cultures grown in different carbon sources. MMM= molecular mass marker; Suc = sucrose; Fru = fructose; Glc = glucose; Xyl = xylose; Asc = ascorbic acid; Gal = galactose; Man = mannose; Cel = cellobiose. The corresponding GTF is tentatively identified in the figure by its molecular mass.

We analyzed the produced enzymes in each carbon source through zymograms to explore closely the strain performance. As shown in Figure 2, in sucrose cultures, four bands are obtained in the zymogram, the upper band corresponding to the 308 kDa dextransucrase (defined as DsxD); two activity bands of around 180 kDa corresponding to dextransucrases DsrD, and DsrI, and a fourth activity band of around 130 kDa, associated to levansucrases LevC, LevL, and LevX, which have very similar molecular mass. Surprisingly, when comparing the zymogram profile obtained from cultures with different carbon sources, differences and similarities in the band pattern are observed, in particular, the lack of the DsxD band in cultures with xylose (Xyl) and ascorbic acid (Asc). These compounds, unlike the other carbon sources, share a particular carbohydrate metabolic pathway: the pentose interconversion.

**Figure 3:**
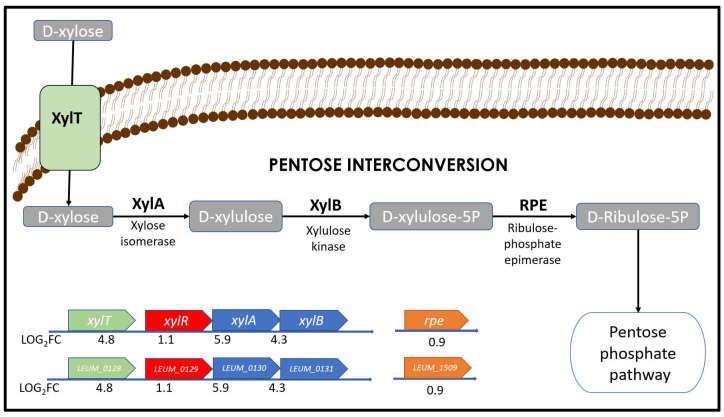
Xylose assimilation and its metabolism in the pentose interconversion. Only xylose uses this metabolic pathway from among the CS tested. Up-regulated genes when using xylose: *xylT* (corresponding to symporter) and the operon *xylAB* (corresponding to xylose isomerase XylA, and xylulose kinase XylB). XylR is the regulatory element that effects over *xylT*, *xylA* and *xylB*.

In the pentose interconversion pathway (Figure 3), D-xylose is isomerized and phosphorylated in successive reactions catalyzed by the xylose-isomerase *xylA* (LEUM_0130), and the xylulokinase *xylB* (LEUM_0131) to produce xylulose-5P a common metabolic intermediate that can be then converted to D-ribulose-5P and enter the pentose phosphate pathway [54]. Although the regulatory pathway for GTFs in *Leuconostoc* strains is poorly understood, a preliminary conclusion regarding the expression of DsxD can be made, considering that its regulation in xylose and ascorbate could be related to the pentose interconversion pathway.

### 3.2. Differential Expression Analysis (DEA): Using Xylose Produces Major Changes in Gene Expression

To explore in detail GTF induction under common carbon sources, we studied how fructose and xylose affect gene expression compared to sucrose as the control condition. As far as we understand, this is the first DEA performed in a *Leuconostoc* wild-type strain, regarding the GTFs genes’ response to carbon sources. Since any single modification in bacterial culture can affect gene expression, we established a rather large cut-off for the Fold-Change in level expression (FC = 4), to narrow the final number of Differentially Expressed Genes (DEGs) to analyze. Therefore, we considered that if LOG_2_(FC) > 2 or <−2, then the gene is up-regulated or down-regulated, respectively. This cut-off permits us to observe the most dramatic changes in gene expression when using xylose and therefore, to make the first approach to understand the regulation in the strain.

**Figure 4:**
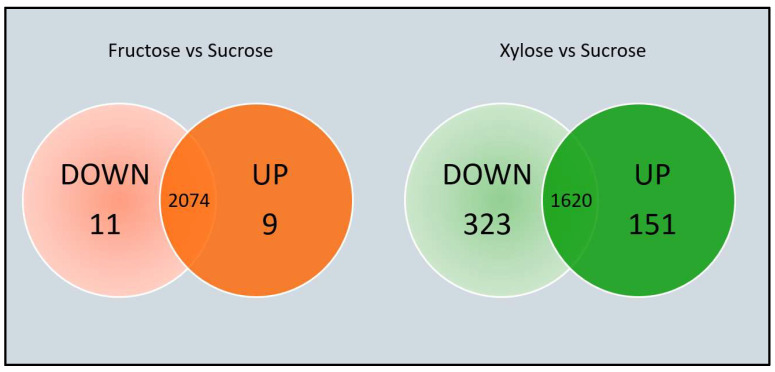
Venn diagram representing differentially expressed genes (DEGs) in *Ln. mesenteroides* ATCC 8293 grown under three different carbon sources. Orange = Fructose as compared to Sucrose; Green = Xylose as compared to Sucrose. The number of Up- and Down-regulated genes is indicated in the diagram. The greatest number of affected genes occurs when xylose instead of sucrose is used as a carbon source.

When comparing the number of DEGs obtained in cultures with fructose or xylose as carbon source instead of sucrose, we observed the greatest effect for xylose, with a small number of genes affected when fructose was used as carbon source (Figure 4). As mentioned earlier, this is most probably the consequence of the xylose metabolism through the pentose interconversion pathway.

*Leuconostoc mesenteroides* bacteria are selective in terms of the carbohydrate used as a carbon source; hence, there are specific carbohydrate transport systems classified in their soft-core genome [24]. Unfortunately, these systems are still poorly characterized so, for instance, in the *Ln. mesenteroides* ATCC 8293 annotated genome, 31 genes are identified as “generic transporters belonging to the Major Facilitator Superfamily (MFS)”. In unpublished experimental results obtained in our group, we speculated that sucrose, glucose, and fructose could be transported into the cell by the mannose PTS. However, when examining the transporters expression level, we found one up-regulated membrane transporter when using fructose (LEUM_0853) and a different one up-regulated in xylose (LEUM_0128). These findings indicate that fructose and xylose enter the cell through membrane symporters from the MFS.

As expected, genes related to xylose transport and metabolism are highly up-regulated when *Ln. mesenteroides* ATCC 8293 uses xylose as CS (Figure 3). These genes correspond to the xylose symporter *xylT* (LEUM_0128, LOG_2_FC = 4.8), the xylose-isomerase *xylA* (LEUM_0130, LOG_2_FC = 5.9), the xylulokinase *xylB* (LEUM_0131, LOG_2_FC = 4.3). It is most probable than *xylA and xylB* genes constitute the *xylAB* operon, since their expression level is similar in this condition. The transcriptional factor *xylR* (LEUM_0129, LOG_2_FC = 1.14) has a repressor effect on genes *xylT, xylA, and xylB* according to RegPrecise database [55], and, therefore, their expression level is different compared to neighbor genes.

In Table 1 we observe a clear decrease in the expression level of all GTF genes when fructose or xylose are used instead of sucrose as carbon source, supporting the established hypothesis that sucrose is the strongest expression inductor. These results are also consistent with the overall decrease in total activity reported in Figure 1, more evident in xylose than in fructose cultures. Similarly, these results match the qualitative observation from the image analysis of the zymogram in Figure 2, where the intensity of the activity bands corresponding to the sucrose culture sample is rather similar to that of the fructose sample but dimmer in the xylose culture activity bands. Since we used the same amount of total protein for the zymograms, together with the transcriptomic results, we may conclude that dimmer bands correspond to less enzyme quantity and activity, therefore, less expression. A deeper analysis of the *Ln. mesenteroides* ATCC 8293 genome could provide complementary information regarding operons and regulatory boxes that can be used in future research to expand the regulatory network. We think that additional biochemical and molecular approaches, such as pull-down and DNA affinity chromatography, must be used to prove these findings.

In an overall view of GTF expression in cultures containing fructose as carbon source, an interesting observation may be derived based on our original hypothesis. In effect, there is an overall decrease in the expression level of all GTF genes when fructose is used as the carbon source, but with the largest effect in *levX*, *levL*, and *levC* the fructansucrases genes. This may result in a lower content of the overall fructansucrase activity, and eventually, a lower amount of fructans compared to glucans if sucrose is available for polysaccharide synthesis after the fermentation process carried out with fructose as carbon source. Eventually, this may also become a strategy to direct cultures of this strain towards the synthesis of diverse fructan-free glucans.

### 3.3. Exploring Possible Regulation Pathways of Leuconostoc mesenteroides ATCC 8293 GTFs

Although sucrose has been generally recognized as the inductor in GTFs expression in *Leuconostoc* strains, the mechanism behind GTFs regulation has yet to be demonstrated [22]. Very few works deal in detail with the eventual physiological mechanism; instead, most reports related to regulation limit their scope to report the differences in total GTF activity in response to environmental changes, such as carbon source [21], temperature [56,57], or oxygen availability [58,59]. D-xylose is a major component of cell walls in plants, and therefore, a carbon source for *Leuconostoc* bacteria since their ecological niche is vegetal matter and roots [3]. Based on the large differences in gene expression between cells grown in D-xylose compared to sucrose cultures (xylose/sucrose), we carried out a detailed analysis of these differences as inferred from the known regulatory pathways in Firmicutes species in search of genomic clues of GTFs regulation.

One of the most down-regulated genes found when using D-xylose is *pgi* encoding a glucose-6-phosphate isomerase, involved in exopolysaccharide (EPS) production in Lactic Acid Bacteria (LAB) species. This is the case for instance of *Lactococcus lactis* and *Strep. thermophilus* where Pgi produces hexose-phosphate as the substrate to synthesize sugar nucleotides, the EPS precursors [60]. However, these are EPS involving Leloir GTFs in their synthesis, requiring high-energy activated sugars as substrate for transglycosylation [17]. Consequently, their regulation can be related to the energy metabolism and *pgi* expression. Considering the zymogram results, it is possible to relate *pgi* with regulation, at least for the *dsxD* gene. Since *pgi* is regulated by the transcription factor CcpA (Carbon catabolite control protein A), we first explored this regulation element.

### 3.4. Genomic Context of GTFs Genes

**Figure 5:**
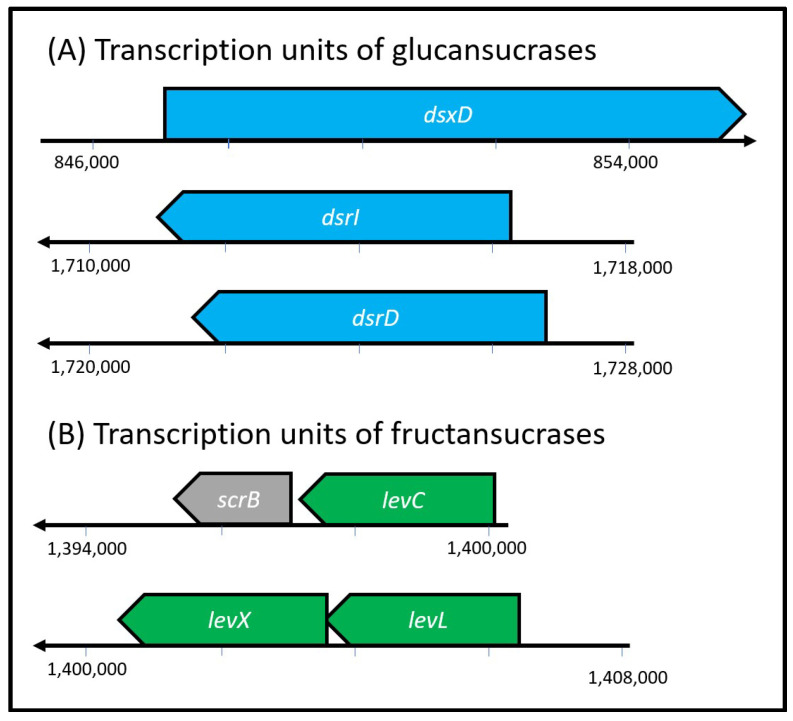
Arrangement of GTFs genes in transcription units. While the glucansucrases genes (**A**) are scattered throughout the genome, the fructansucrase genes (**B**) form two operons.

The total length of the sequenced genome of the *Ln. mesenteroides* ATCC 8293 strain is 2.07 Mb. It consists of 2047 genes including the GTFs genes. When analyzing the localization of the GTFs genes in the strain genome, we observed that the glucansucrases genes (*dsxD*, *dsrD*, and *dsrI*) are scattered through the genome, and as concluded through the Operon Mapper software analysis [47] these genes are neither located in operons (Figure 5A). On the other hand, the fructansucrase genes are located in the negative strand, within the same region in the genome (Figure 5B) and arranged in two operons: *levLX* and *levC*-*scrB*. The *scrB* gene also encodes a sucrose-6-phosphate hydrolase. Given that fructansucrases are arranged in operons, we used the upstream intergenic region of operon *levLX* and *levC*-*scrB* for the region analysis.

### 3.5. The Transcription Factor (TF) CcpA

The protein CcpA plays a central role in the regulation of the Phosphoenolpyruvate-dependent Transferase System (PTS), the main pathway for carbohydrate uptake in bacteria [61] and controls carbohydrate metabolism in several species. CcpA belongs to the LacI-GalR family of bacterial TFs which represses gene expression by allosteric hindrance [62]. CcpA forms a complex with phosphorylated HPr to be able to bind the gene promotor region preventing transcription. The DNA sequence where the complex CcpA-Hpr-P binds to the promotor is known as the catabolite response element (*cre*), and its canonical sequence has been identified for *Lactobacillaceae* (WTGWAARCGYTTWCAW) through experimental and comparative genomics [55,61]. Previously, in unpublished research of our group, we were not able to locate the *cre* binding site within the promoter region of GTFs from *Ln. mesenteroides* ATCC 8293, discarding the participation of CcpA in GTFs regulation. To update our findings, we analyzed the upstream intergenic region of each gene with the FIMO tool from MEME-suite [49,50] in a more detailed search for the canonical *cre* site. Again, the *cre* binding site was not located. Hence, we may conclude that in the strain *Ln. mesenteroides* ATCC 8293, the GTFs genes are not regulated by CcpA. Furthermore, when analyzing the transcription levels of the PTS components in our differential expression analysis on xylose/sucrose, we found that the genes: *ccpA* (LEUM_0544, LOG_2_FC = −1.2), *ptsH* (corresponding to HPr protein; LEUM_1780, LOG_2_FC = −1.45), and *scrA* (corresponding to the phosphate-kinase; LEUM_0508, LOG_2_FC = −3.7) are down-regulated, suggesting a minor quantity of CcpA protein and thus, limiting their activity as repressors under these culture conditions.

### 3.6. The Transcription Factor PerR

Recent work suggests that GTFs expression in *Ln. mesenteroides* BD3749 strain responds to the environmental oxygen level; since expression of the GTF, identified as Gsy in this strain, increases as a consequence of Reactive Oxygen Species (ROS) accumulation, the synthesized polymer may then act as cell protector absorbing ROS [59]. These results allow us to speculate that a key to GTFs regulation could be ROS since *Leuconostoc* strains lack catalase, requiring the H_2_O_2_ sensor PerR to deal with oxidation stress. PerR is a dimeric protein that in its metal-bound form binds DNA, preventing the expression of the regulated genes; when H_2_O_2_ levels rise, a series of reactions are triggered, which induces a conformational change in the protein, preventing binding to DNA [63].

In our DEA, perR (LEUM_0492) is down-regulated in xylose/sucrose (LOG_2_FC = −2.3), limiting its activity as a repressor, as already described for CcpA. Furthermore, we analyzed growth and total GTF activity in cultures obtained in anaerobic conditions compared to aerobic cultures. Contrary to results reported for *Ln. mesenteroides* BD3749, *Ln. mesenteroides* ATCC 8293 does not show differences in total GTF total specific activity or growth in cultures obtained either in aerobic or anaerobic conditions (Table 2). Furthermore, in an additional report dealing with *Ln. mesenteroides* YL48 the authors claim that, in terms of regulation response to ROS, GTF up-regulation occurs in oxygen-depleted cultures with high CO_2_ levels [58]. The ensemble of results regarding ROS response suggests that the regulation of GTFs can be particular for each strain.

### 3.7. The Transcription Factor LexA

Recent work suggests that GTFs in *Weissella cibaria* C2-32 may be regulated by TF LexA, a protein involved in the SOS response system [57]. In *Escherichia coli* growing in normal conditions, dimeric LexA binds to the DNA strand and represses DNA repair genes; meanwhile, in severe culture conditions, such as low pH-, LexA forms aggregates with a lower capacity to repress transcription [64]. In our transcriptomic data, lexA in xylose/sucrose was up-regulated (LEUM_1204, LOG_2_FC = 2.4), while its expression remains similar in fructose/sucrose (LEUM_1204, LOG_2_FC = 0.1). This led us to search for the LexA binding site sequence in the GTFs upstream regions using the FIMO tool [49], finding one match in the upstream sequence of the *levLX* operon. These preliminary findings suggest that *levLX* operon is repressed by LexA when *Ln. mesenteroides* grows on xylose as a carbon source. Interestingly, the final pH, reached in the stationary phase is lower when using sucrose (pH = 4.53), a bit higher in fructose (pH = 5.26), and even higher in xylose (pH = 6.13). Nevertheless, this implies that the low pH in the final stage of the culture may become a severe condition for the strain ATCC 8293, affecting LexA’s ability to bind to DNA to allow the transcription of the *levLX* operon.

### 3.8. VicK/VicR Two-Component System

There is an enormous interest in understanding the regulation of GTFs in *Streptococccus mutans* since these enzymes allow the bacteria the synthesis of the biofilm that enables cell adhesion to teeth, the initial step that leads to dental plaque formation and caries in humans [65]. This biofilm is mainly formed by polysaccharides synthesized by GTFs from sucrose, so their activity is considered a virulence factor [19,20]. GTFs enzymes from *Strep. mutans* and *Ln. mesenteroides* are orthologous proteins with high sequence identity (48%); as both species belong to the *Lactobacillales* order, they may share regulatory pathways. In *Strep. mutans*, the two-component system *vicRK* (formerly *covRK*) regulates the transcription of *gtfBCD* operon (where GTFs genes are located) in response to temperature stress—VicK auto-phosphorylates at high temperatures, transferring a phosphate group to VicR, that then binds to the promoter region of regulated genes to repress its expression [66]. Searching in the DEA, we found that the level of expression of the *vicRK* system remains constant in our experiments and comparisons (xylose/sucrose and fructose/sucrose). Consequently, we concluded that *vicRK* is not responsible for the observed changes in GTFs expression in cultures grown with different carbon sources. However, when we searched for the VicR binding site TGTWAHNNNNNTGTWAH [51] (where W is A or T and H is A, T, or C) in the GTFs intergenic upstream sequences using FIMO, we obtained regions for possible binding sites in the fructansucrases operons (Table 3). These findings suggest that fructansucrases genes regulation may be affected by temperature stress, another adverse condition that must be explored to understand their role in GTFs regulation.

### 3.9. Transcriptomics Validation by Retro-Transcriptase qPCR

We validated our transcriptomics results with a qPCR analysis of the GTFs with the *dnaX* gene as housekeeping (data in Supplementary Materials File S3 and File S4). We used fructose as the control condition since we observed no significant difference in *dnaX* expression between fructose and sucrose, and also because no EPS is produced in cultures with fructose as CS. In general, the RT-qPCR showed the same expression pattern found in the transcriptomics analysis: *levC*, *levX*, *levL*, *dsrI*, and *dsxD* are down-regulated, while there is no significant difference (*t*-test with α = 0.05) in *dsrD* expression between the conditions essayed (Table 4). As a result of the RT-qPCR experiments, we observed a consistent down-regulation in GTFs expression when modifying the CS, as already observed through transcriptomics, validating the former.

## 4. Conclusions

The scarce information concerning GTF regulation makes it difficult to assemble a possible general regulatory network. We contribute to the task by analyzing known regulatory pathways from GTFs Firmicutes producers in the context of our DEA experiments. A deeper analysis of the *Ln. mesenteroides* ATCC 8293 genome could provide additional information regarding operons and regulatory boxes that may be used in new searches to expand the knowledge regarding the regulatory network. The availability of nutraceutical bioactive polysaccharides during fermentation through regulation of GTFs expression may enhance the symbiotic properties of characteristics of fermented foods. This in turn may be achieved by controlling certain aspects of traditional food fermentations such as temperature, pH, carbon availability, or oxygen content. Additional biochemical and molecular approaches, such as pull-down and DNA affinity chromatography, together with adverse environmental conditions can be the key to understanding the regulation of GTFs and must be used to validate our findings. More regulation information requires the expression quantification (qPCR) and production of each GTF (quantitative proteomics) in cultures obtained from different carbon sources, exploring also the effect of additional culture conditions such as temperature, pH, oxidative stress (oxygen, H_2_O_2_, and CO_2_ effect), in this and additional strains bearing diverse GTFs genes.

## Figures and Tables

**Table 1:** Change in the expression level of GTFs *Ln. mesenteroides* ATCC 8293 with sucrose as control.

ID Locus	Gene	LOG_2_ FC (Xyl/Suc)	Xylose	LOG_2_FC (Fru/Suc)	Fructose
LEUM_1409	*levC*	−2.726	Down-regulated	−1.442	NCD *
LEUM_1410	*levX*	−2.135	Down-regulated	−0.506	NCD *
LEUM_1411	*levL*	−2.813	Down-regulated	−0.500	NCD *
LEUM_0857	*dsxD*	−2.059	Down-regulated	−0.081	NCD *
LEUM_1747	*dsrD*	−0.616	NCD *	−0.193	NCD *
LEUM_1752	*dsrI*	−2.220	Down-regulated	−0.149	NCD *

NCD * No Change Detected with cut-off LOG_2_(FC) > 2 or <−2.

**Table 2:** Total specific activity and OD in stationary phase in anaerobic and aerobic cultures.

Condition	Total Activity (U/mg Total Protein)	Biomass (OD_600 nm_) Reached
Aerobic	1.06 ± 0.1	6.5 ± 1.2
Anaerobic	1.04 ± 0.1	6.3 ± 0.9

**Table 3:** FIMO matches for vicR binding site in the upstream regions of levansucrases genes.

Sequence Name	Strand	Start	End	*p*-Value	q-Value	Matched Sequence
Upstream *levC-scrB*	negative	90	106	2.81 × 10^−5^	0.118	tgtttcctctctgttaa
Upstream *levLX*	positive	408	424	8.97 × 10^−5^	0.138	tgtaatactattgaaat

**Table 4:** Comparison of relative gene expression in RNA-seq and RT-qPCR of GTFs.

		RT-qPCR	RNA-Seq
ID	Gene	LOG_2_FC Xyl/Fru	LOG_2_FC Xyl/Fru
LEUM_1409	*levC*	−3.73	−1.67
LEUM_1410	*levX*	−4.03	−2.01
LEUM_1411	*levL*	−3.53	−2.69
LEUM_0857	*dsxD*	−6.83	−2.24
LEUM_1747	*dsrD*	−1.61	−0.93
LEUM_1752	*dsrI*	−4.71	−2.45

## Data Availability

Data is contained within the Supplementary Material.

## Supplementary Materials

The following supporting information can be downloaded at: https://www.mdpi.com/article/10.3390/foods12091893/s1, Table S1: Primers used for qPCR. File S1: transcript abundances and LOG_2_FC for xylose/sucrose conditions. File S2: transcript abundances and LOG_2_FC for fructose/sucrose conditions. File S3: Ct raw data from the qPCR experiment. File S4: ∆∆Ct calculations for the qPCR experiment.
